# The Institutional Library

**Published:** 1914-02-07

**Authors:** 


### THE INSTITUTIONAL LIBRARY.

Manual of Bacteriology. By R. Muir, M.A., M.D., and James Ritchie, M.A., M.D. Sixth Edition. (London: Henry Frowde and Hodder and Stoughton. 1913. Price 10s. 6d.)

Since the first edition of this manual was brought out in 1897 by Young J. Pentland, bacteriology has literally evolved at a steady rate—recently at an accelerated pace; it is not uninteresting, therefore, to watch the progress of such a manual as this, which long ago obtained the position of a standard work. Already in the preface to the first edition reference is made to the extensive growth of the science of bacteriology. Yet how small was the field then to what it is now! Concurrently with this advance the authors and publishers of this manual have kept abreast of the times, and the present sixth edition is not only still probably the best text-book from the students' point of view, but also a most convenient repository of all that is of tried value in this increasingly important science. Suffice it to say that in subject-matter and in illustration the manual more than maintains its position in the forefront of bacteriological text-books. A number of excellent new micro-photographs have been added to this edition, while two new sections are to be found—one on the bacteriology of milk, the other a chapter on pathogenic fungi. On the subject of Hoffmann's bacillus the authors still favour the opinion that this organism is distinct from the diphtheria bacillus, in spite of the recent work done in this direction.

### The Latest Hospital History.

The History of St. George's Hospital. By G. C. Peachey. Parts III. and IV. Price 5s. net.

After a considerable interval since the issue of the first two parts of this history Mr. Peachey now issues the third and fourth parts together, as already noted in The Hospital (January 3, 1914; p. 361). These two parts deal with the history of the hospital during the first twenty years of its existence, while it was still outside the London of the early eighteenth century. They fully maintain the high standard of excellence which was set by the first two parts; they are, in fact, monuments of painstaking research crammed with valuable information about the early history of the hospital. Incidentally, they also throw a flood of light upon the domestic life of that time, and are thus of interest not alone to those who are, or have been, connected with St. George's, but also to all institutional workers, and, indeed, to every London lover too.

To summarise this wealth of incident would require too much space; we can but give a few flowers culled from this old London garden of physic. Thus it appears that the first name of the new institution was the Hyde Park Hospital. A year later a petition to King George II. for a charter was drawn up but never presented; in this petition mention of St. George appears for the first time, probably in deference to the reigning monarch. The parish of St. George's, Hanover Square, had already been so named for a similar reason; the hospital stood within this parish, an additional reason for the name.

It was quite clearly apprehended from the first that the purpose of a voluntary hospital was not to relieve those for whom the Poor-Law existed, but the next higher classes in the social scale. As early as 1741 abuse of the charity was detected. Steps were taken to check it, not by appointing a professional almoner but by individual governors taking it upon themselves to examine the circumstances of those applying for relief. Within three years of the foundation of the hospital it had been extended to contain two hundred beds, and eight years later another fifty were added.

The freehold was purchased in 1736 from the Dean and Chapter of Westminster Abbey for £500; this is interesting in view of the enormous sum, approaching half a million, which Mr. Mallaby-Deeley was arranging to give for the site a few months ago, though it has to be remembered that he was negotiating for land of more than twice the area of the original freehold plot. Another curious land transaction was the purchase in 1749 of three and a half acres in Brompton for use as a burial-ground at a cost of £460 and a fine of £41 17s. 6d. This land was resold in 1825 for the erection of Holy Trinity, Brompton; but Mr. Peachey does not tell us what price it fetched. Incidentally, several of the graves in Holy Trinity Churchyard are of older date than 1825, so possibly others besides deceased inmates of the hospital were allowed to be buried there. Another minute shows that in 1735 a scheme was set on foot for accommodating twenty-five women in the hospital during the last month of pregnancy. It fell through, but evidently St. George's had governors in those days who were nearly two centuries in advance of their times.

### Radium Therapy.

Radium Therapeutics. By N. S. Finzi, M.B. (London: Henry Frowde and Hodder and Stoughton. 1913. Pp. 112. Price 6s. net.)

Short as is the interval since the isolation of radium by Mme. Curie in 1910, an enormous fund of information about the properties of this uncanny substance has been accumulated, and its therapeutic properties have received nearly as full a share of attention as its physical and chemical attributes. The latter are, however, worked out with much more certainty than the former; although undoubtedly our knowledge of radium and its emanations is still very imperfect, it is certain that therapeutically the darkness of our ignorance is much denser.

In this monograph the author begins by a succinct account of the leading properties of radium as established by the latest researches. He deals very fully with the technical details of the storage of radium, the collection of its emanations, the selective screening of the different rays, and, in fact, the expert technique of the application of radium to therapeutics. Finally come chapters on the various diseases for which radium and its products have and have not proved valuable. Naturally, in view of the events of early this year, summarised in a leading article in The Hospital on January 17, p. 410, the sections on malignant disease are of the chief interest. In all essentials Dr. Finzi's opinions coincide with those expressed in that article, and they form a full, able, and temperate statement of the case as it stands at present. Surgical treatment is awarded pride of place still for operable cases, but a plea is entered for combining with it radium therapy before the stage of hopelessness is reached. It is hinted, somewhat obscurely, that for carcinoma of the cervix uteri radium treatment with diathermy is superior to Wertheim's operation. It may turn out to be so, but it could have been wished that the paragraph had been worded with a little more candour.

As a précis of the present position of radium treatment this book is excellent, both in itself and as a landmark. Unfortunately it is certain to be out of date in a year or two, so that its appeal is necessarily greater to the specialist than to the practitioner. To those in charge of radium departments in institutions it will prove invaluable and indispensable.

Diet in Dyspepsia and Other Diseases of the Stomach and Bowels. By William Tibbles, M.D. (London: The Scientific Press, Ltd. 1913. Pp. 150. Price 2s. 6d.)

This text-book is intended primarily for nurses and lay readers, and it may be conceded at once that the subject is treated much more sensibly than many so-called guides on the treatment and pathology of alimentary functions. The book is essentially practical, and, as far as possible, Dr. Tibbles has avoided the difficulty of going too deeply into those conditions, which can only satisfactorily be treated by the medical attendant. Although on several points it would be easy to criticise some of the directions recorded, it must be admitted that these lectures are well worth bringing out in book form. Nurses especially will be glad to have in such a handy and permanent form the lectures with which so many of them are familiar from reading them in *The Nursing Mirror*. It is allowable to utter a protest against the use of the word "mumps," even in a text-book for nurses, when a simple inflammation of the parotid gland is meant, though we may at the same time congratulate Dr. Tibbles on his employment of "parotiditis" for the most usual but less correct "parotitis." A large number of proprietary goods are mentioned more or less impartially, though the inclusion of one or two practically unknown preparations is open to criticism. We must finally draw the author's attention to the objectionable use of the word "lady" on page 36.

### A Modern "De Senectute."

Old Age. By Robert Saundby, M.D. (London: Edward Arnold. 1913. Pp. 312. Price 7s. 6d. net.)

Experience and statistics show that the average duration of life in civilised communities is increasing steadily, and that mental and bodily activity among octogenarians and even older people still is much commoner and greater than it was, even within the memory of persons still alive. How far the tendency will go it is difficult to say, but if medicine and surgery continue to progress as they have done in recent decades, it would seem as if longevity will go on increasing for some generations yet awhile. At any rate, Dr. Saundby has done his best to promote such a state of affairs by compiling a most excellent book on the care and treatment of the old both in health and disease. He starts off well with a most interesting chapter on the duration of life, containing much curious information about noted centenarians, or reputed centenarians, of the past. The list, curiously enough, omits Manuel Garcia, but it includes many wonderful records. Perhaps the most astonishing examples are those collected last year from Russia, when eight contemporaries of the Battle of Borodino were discovered to be alive. One of these was a sergeant-major who is alleged to be one hundred and twenty-two, and another was a woman who asserts that she remembers the Burning of Moscow. Dr. Saundby then deals in turn with all the diseases which afflict mankind, considered as to the way in which they affect the aged. Quite properly he pays great attention to diet and to environment of all kinds; and throughout his chapters he enriches his discourse by apt quotation of actual cases from his experience and by occasional well-told anecdotes. In fact, he has produced a volume which is scholarly without being dull, and is a valuable contribution to medical literature. As he says, there has been no recent attempt to focus the results of modern research as they affect the life of the aged; and that want Dr. Saundby well supplies. His "De Senectute" is not unworthy of the Ciceronian tradition.

### Sanatorium Lectures.

Lectures on Tuberculosis to Nurses. By Oliver Bruce, M.R.C.S., L.R.C.P. Pp. viii + 134. Crown 8vo. (H. K. Lewis. Price 2s. 6d. net.)

Lectures to nurses employed in sanatoriums or as tuberculosis and health visitors form a not unimportant part of the duties of the medical officers attached to institutions for the treatment of tuberculosis. Many nurses employed as visitors in connection with dispensaries have not had the advantage of sanatorium training, and it is therefore important that they should be thoroughly grounded in the hygienic and dietetic measures which they must endeavour to get carried out in the homes they visit. This volume by Mr. Bruce is, however, not likely to be of much value to them in this connection or to those who turn to it in the hope of obtaining hints and suggestions as to lectures. The book consists of six lectures delivered to the Queen Victoria Jubilee Nurses, but it is difficult to imagine that some of the subjects discussed by the author can be of much use to those for whom the volume is intended. The technique of the method for estimating the opsonic index, and the indications and contra-indications for the use of tuberculin and its dosage are subjects which scarcely come within the purview of tuberculosis nurses. In his second lecture the author mentions the various causes of cough and then says: "I will endeavour to show you how to differentiate between them and a cough due to tuberculosis;" and, again, "new growths you will never, I think, find a stumbling-block." Surely Mr. Bruce does not consider that it is the duty of a nurse or tuberculosis visitor to make a differential diagnosis, though the tenor of these and other remarks certainly gives one that impression. The author's expression is at times loose and inaccurate. He speaks of the Tuberculosis Bill when he means the National Insurance Act, and imagines that the compulsory notification of all cases of tuberculosis is one of the provisions of that Act, instead of being due to an Order by the Local Government Board.

Cancer of the Breast. By C. B. Lockwood, F.R.C.S., Consulting Surgeon of St. Bartholomew's Hospital. (London: The Oxford University Press. 1913. Pp. 234. Price 10s. 6d. net.)

This book reflects very strongly the individuality and personality of its author. It is not, as the title might lead one to suppose, a systematic treatise on cancer of the breast, but a record of Mr. Lockwood's own experiences, operations, and results. This, we feel, is a drawback, for the dogmatic personal note, so usefully struck by Mr. Lockwood in his clinical teaching of students at St. Bartholomew's Hospital, is of much less value in a monograph designed for practitioners and surgeons. Mr. Lockwood's experience has been so extensive, and his powers of observation are so acute, that interesting cases, practical hints based upon them, and many more sidelights upon the obscurities of breast cancer abound. Nevertheless, one leaves the book with a slight sense of disappointment; for, good as it is, one feels that Mr. Lockwood could write a better. Even his own operative results are nowhere tabulated or analysed statistically in full. And half-a-guinea seems an excessive price for a by no means large book with so few words to a page as this one has.

